# 1316. Uncommon Presentations of Common Variable Immunodeficiency

**DOI:** 10.1093/ofid/ofab466.1508

**Published:** 2021-12-04

**Authors:** Akankcha Alok, John Greene, Sadaf Aslam

**Affiliations:** 1 H. Lee Moffitt Cancer Center, Wilmington, Delaware; 2 Moffitt Cancer Center, Tampa, FL; 3 University of South Florida, Tampa, Florida

## Abstract

**Background:**

Common Variable Immunodeficiency (CVID) is a primary immunodeficiency disorder which affects B lymphocyte function and differentiation causing decreased levels of Immunoglobulin G (IgG), Immunoglobulin A (IgA) and Immunoglobulin M (IgM).^1^ The objective of this study is to highlight how hypogammaglobulinemia can lead to respiratory infections with microbes that are lesser known in the background of CVID with the help of a two-case series.

**Methods:**

Medical records of two patients with CVID were reviewed who were found to have mycobacterium avium-complex intracellulare and streptococcus agalactiae lung infections respectively.

**Results:**

Decreased IgG in CVID means reduced antibody production, low IgA leads to mucosal inflammation and increased susceptibility to respiratory infections^2^ and lower IgM memory B-cells causes infections with encapsulated microorganisms.^3 ^Table 1 highlights the various respiratory infections and their etiologies that have been reported with CVID, the most common being encapsulated organisms like Haemophilus influenza, Streptococcus pneumonia, Neisseria meningitidis along with enterovirus. Table 2 demonstrates our findings. In the first case we have reported a patient with mycobacterium avium-complex intracellulare (MAC-I). This could be because of hypogammaglobulinemia, decreased B and T-cell interaction and reduced T-cell signaling caused by CVID.^4 ^ Although, mycobacterium tuberculosis, simiae and hominis lung infections and mycobacterium bovis systemic infections have been reported before, MAC-I is relatively rare in CVID.^5^ In our second case, the patient developed streptococcus agalactiae or Group-B streptococcus (GBS) empyema. Most cases of GBS have been reported in pregnant women and infants. Infections with other encapsulated organisms have been reported in CVID but GBS empyema is less frequent and can happen due to decreased bacteria-specific CD4 cells, microbial translocation and hypogammaglobulinemia.^6 .^

Table 1. Respiratory Infections reported in CVID along with their etiologies.

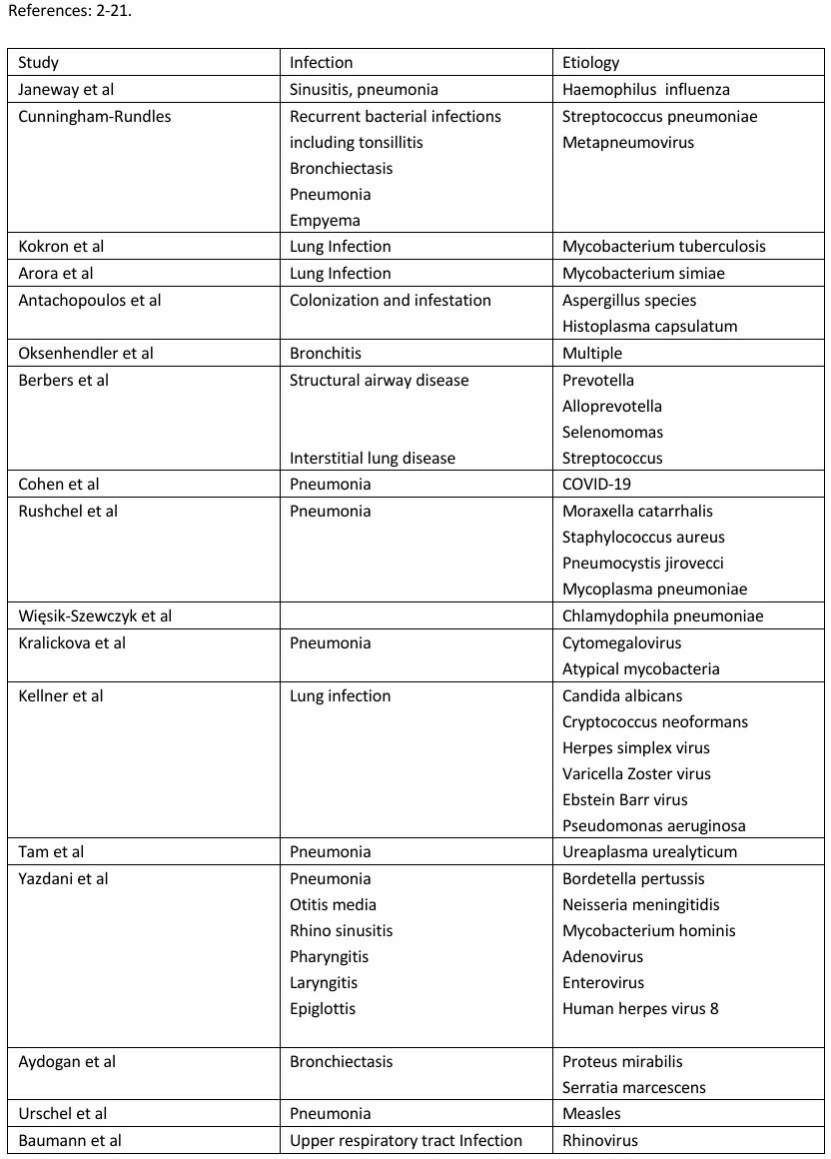

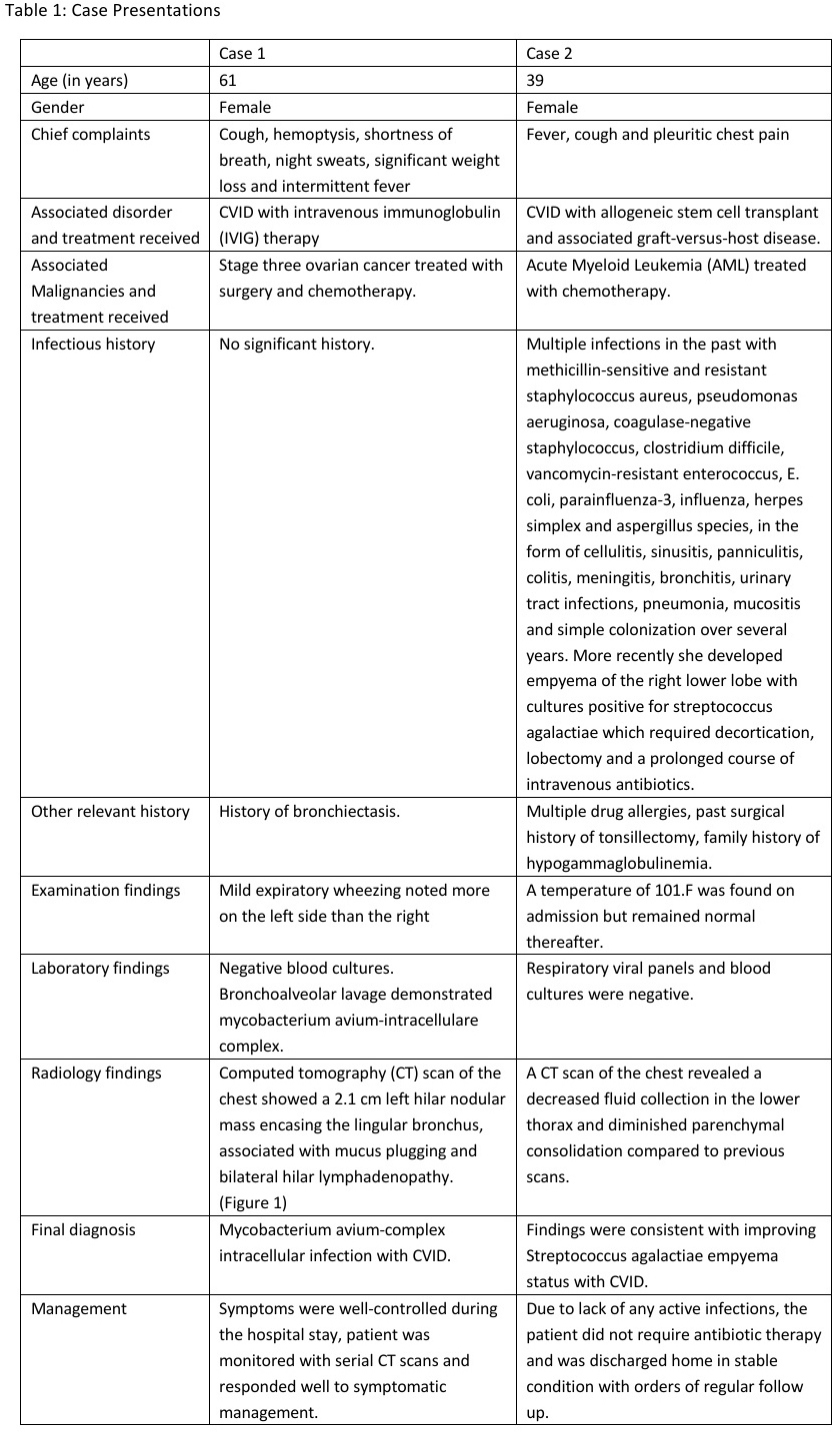

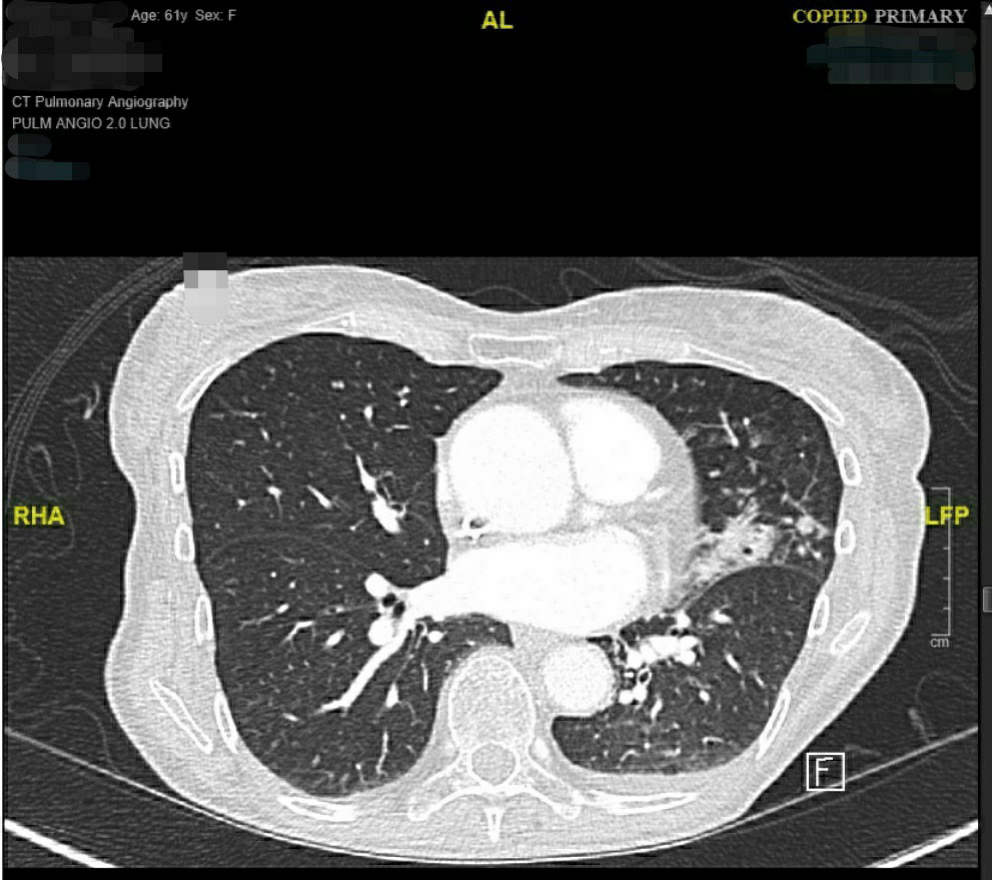

Figure 1. CT image of MAC-I infection.

**Conclusion:**

We encountered two unique cases of CVID with rare infectious etiologies. The cases are intended to create an awareness and vigilance regarding CVID induced hypogammaglobulinemia which can cause respiratory infections with lesser known pathogens where antibodies may be important.

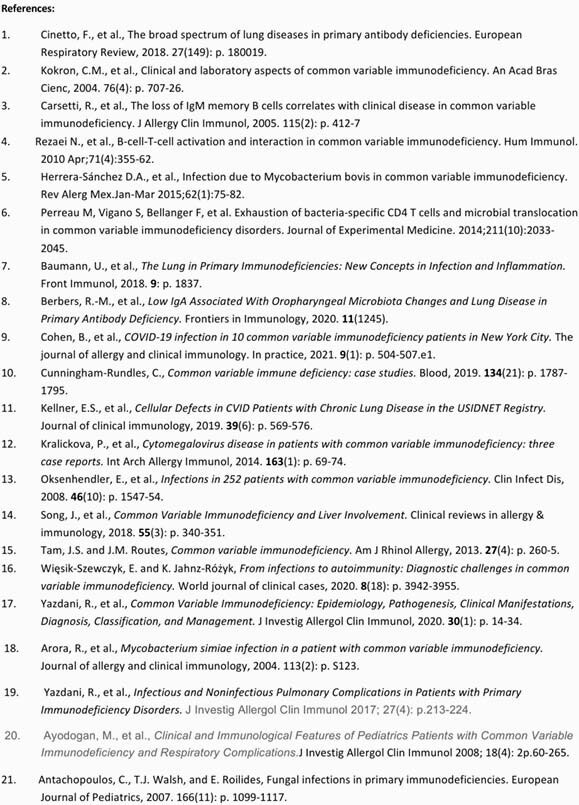

**Disclosures:**

**All Authors**: No reported disclosures

